# Factors Associated with Preferences for Long-Acting Injectable Antiretroviral Therapy Among Adolescents and Young People Living with HIV in South Africa

**DOI:** 10.1007/s10461-022-03949-2

**Published:** 2023-01-09

**Authors:** Elona Toska, Siyanai Zhou, Jenny Chen-Charles, Lesley Gittings, Don Operario, Lucie Cluver

**Affiliations:** 1 Centre for Social Science Research, University of Cape Town, Cape Town, South Africa; 2 Department of Sociology, University of Cape Town, Cape Town, South Africa; 3 Department of Social Policy and Intervention, University of Oxford, Oxford, UK; 4 School of Public Health and Family Medicine, University of Cape Town, Cape Town, South Africa; 5 School of Health Studies, Faculty of Health Sciences, Western University, London, Canada; 6 Rollins School of Public Health, Emory University, Atlanta, USA; 7 Department of Child and Adolescent Psychiatry, University of Cape Town, Cape Town, UK; 8 Centre for Social Science Research, Leslie Social Sciences Building, University of Cape Town, 4.89, Rondebosch, Cape Town, 7700 South Africa

**Keywords:** Adolescents, Antiretroviral, Long-acting, Injectables, South Africa

## Abstract

Long-acting injectable anti-retroviral therapy (LAART) may overcome barriers to long-term adherence and improve the survival of adolescents and young people living with HIV (AYLHIV). Research on the acceptability of LAART for this age-group is limited. We asked 953 AYLHIV about their preferred (theoretical) ART mode of delivery (pill, injectable, or other) in 2017–2018, before LAART was available or known to AYLHIV in South Africa. One in eight (12%) AYLHIV preferred LAART over single or multiple pill regimens. In multivariate analyses, six factors were associated with LAART preference: medication stock-outs (aOR = 2.56, 95% CI 1.40–4.68, p = 0.002), experiencing side-effects (aOR = 1.84, 95% CI 1.15–2.97, p = 0.012), pill-burden (aOR = 1.88, 95% CI 1.20–2.94, p = 0.006), past-year treatment changes (aOR = 1.63, 95% CI 1.06–2.51, p = 0.025), any HIV stigma (aOR = 2.22, 95% CI 1.39–3.53, p ≤ 0.001) and recent ART initiation (aOR = 2.02, 95% CI 1.09–3.74, p = 0.025). In marginal effects modelling, 66% of adolescents who experienced all factors were likely to prefer LAART, highlighting the potential high acceptability of LAART among adolescents and young people living with HIV struggling to adhere and have good HIV treatment outcomes. Adolescent boys who reported high ART pill burden were more likely to prefer LAART than their female peers in moderation analyses, suggesting that LAART may be particularly important to improve treatment outcomes among male AYLHIV as they become older. Adding LAART to existing treatment options for AYLHIV, particularly higher risk groups, would support AYLHIV to attain and sustain viral suppression—the third 95, and reduce their risk of AIDS-related mortality.

**Supplementary Information:**

The online version contains supplementary material available at 10.1007/s10461-022-03949-2.

## Resumen

La terapia antirretroviral inyectable de acción prolongada (TAR LA) puede superar las barreras a la adherencia y mejorar la supervivencia de los adolescentes y jóvenes que viven con el VIH (AJVVIH). La investigación sobre la aceptabilidad del TAR LA para este grupo de edad es limitada. Preguntamos a 953 AJVVIH sobre su modo preferido (teórico) de administración de ART (píldora, inyectable u otro) en 2017–2018, antes de que TAR LA estuviera disponible o fuera conocido por los AJVVIH en Sudáfrica. Uno de cada ocho (12%) AJVVIH prefirió TAR LA sobre los regímenes de píldoras simples o múltiples. En los análisis multivariantes, seis factores se asociaron con la preferencia de TAR LA: agotamiento de la medicación (odd ratio ajustada [ORa] = 2,56, IC95% 1,40–4,68 p = 0,002), experimentar efectos secundarios (ORa = 1,84, IC95% 1,15–2,97 p = 0,012), carga de píldoras (ORa = 1. 88, IC95% 1,20–2,94 p = 0,006), cambios de tratamiento en el último año (ORa = 1,63, IC95% 1,06–2,51 p = 0,025), cualquier estigma del VIH (ORa = 2,22, IC95% 1,39–3,53 p ≤ 0,001) y el inicio reciente del TAR (ORa = 2,02, IC95% 1,09–3,74 p = 0,025). En la modelización de efectos marginales, el 66% de los adolescentes que experimentaron todos los factores eran propensos a preferir la TAR LA, lo que pone de relieve la alta aceptabilidad potencial de la TAR LA entre los adolescentes y los jóvenes que viven con el VIH que luchan por adherirse y tener buenos resultados en el tratamiento del VIH. Los adolescentes varones que informaron de una alta carga de píldoras para el tratamiento antirretroviral eran más propensos a preferir la TAR LA que sus pares mujeres en los análisis de moderación, lo que sugiere que la TAR LA puede ser particularmente importante para mejorar los resultados del tratamiento entre los hombres que viven con el VIH a medida que crecen. La adición de la TAR LA a las opciones de tratamiento existentes para las personas que viven con el VIH, en particular los grupos de mayor riesgo, ayudaría a las personas que viven con el VIH a alcanzar y mantener la supresión vírica -el tercer 95- y a reducir el riesgo de mortalidad relacionada con el sida.

## Introduction

Persisting high rates of new HIV infections in the 15–24-year-old age group [1], coupled with improved survival among children who were acquired HIV vertically contribute to a considerable cohort of adolescents and young people living with HIV (AYLHIV) [2]. This large cohort of AYLHIV is critical to reaching the 95–95–95 goals, particularly in sub-Saharan Africa where 88% of them live [3]. The 95–95–95 goals were set by the Joint United Nations Programme on HIV/AIDS (UNAIDS), with aims to: diagnose 95% of all individuals living with HIV; provide antiretroviral therapy (ART) for 95% of all individuals diagnosed; and achieve viral suppression for 95% of those who are on HIV treatment by 2030 [4]. Although AIDS-related deaths decreased among all other age groups, AIDS-related mortality increased by 45% among adolescents and young people between 2005 and 2015 [5]. AYLHIV have the worst HIV outcomes compared to other age groups of people living with HIV [6–9], including high treatment disruption and drug resistance and low rates of viral suppression [10–14].

Oral antiretroviral therapy (ART) has had substantial impacts on the prevention and treatment of HIV, but it requires life-long daily administration [15, 16]. Insufficient adherence is a major barrier to ending AIDS as it leads to drug resistance and treatment failure [17]. AYLHIV, in particular, face significant challenges in ART adherence due to various complex and inter-related factors: the shift in personal responsibility when they transition into adult health services from paediatric health services [18], lack of disclosure [8], HIV-related stigma [2, 19], relational factors, including caregiver/familial support and caregiver changes due to parental mortality [20], poor healthcare quality, and structural factors including stock outs and limited access to facilities [21]. Subsequently, AYLHIV have significant challenges in ART adherence and higher risk of viral failure, risk of HIV exposure among their sexual partners, and AIDS-related mortality and morbidity [22–24]. Interventions to improve ART adherence and long-term HIV outcomes among AYLHIV are vital to help advance progress to end AIDS by 2030 [25].

A growing body of evidence highlights several promising behavioural, psychosocial and healthcare system interventions to improve adherence to oral ART among AYLHIV [26, 27]. However, even when delivered with high efficacy and at scale, such interventions cannot reach all AYLHIV. Additional biomedical solutions acceptable to AYLHIV are needed to augment behavioural and psychosocial interventions. Data from Phase 3 clinical trials testing injectable long-acting ART (LAART) with adult participants have shown this mode of delivery to be non-inferior and a promising alternative that offers advantages over daily oral ART for the treatment and prevention of HIV [28–32]. LAART has the potential to help overcome the obstacles to adherence to daily oral ART among people living with HIV [33]. A recent study on adult female sex workers living with HIV from Tanzania and the Dominican Republic revealed significant positive attitudes towards LAART [33]. It was perceived to lighten the practical and psychosocial burdens of daily pill burden and stigma associated with pill-taking, as well as barriers specific to sex-workers such as their work schedules, mobility and alcohol use [33]. LAART may also reduce the impact of food insecurity as an impediment to daily oral ART adherence [34, 35], as it eliminates the need to take food with pills [36]. Furthermore, LAART could improve access to medication and reduce systemic toxicities [17, 29, 37–39]. A recent study modelling the impact of LAART on AYLHIV in Kenya also showed the potential of LAART to cost-effectively avert AIDS-related morbidity and mortality [40].

During clinical trials in the US, Canada and European countries, adult participants reported significantly higher satisfaction with LAART as well as preference over daily ART [41–45]. Outside of clinical trials, significant interest has also been demonstrated in acceptability studies [30, 46–48]. Compared to daily oral ART, people living with HIV found LAART more convenient and easier [41–43, 45, 49], gave them an increased sense of normality and freedom during their daily lives [42, 43, 46, 48], and created an opportunity to customise ART treatment according to individual needs [50]. Furthermore, LAART was considered to improve confidentiality for patients and subsequently lead to less discrimination. By eliminating the need for daily pills, LAART also reduced internalised stigma and helped to avoid the daily reminder of HIV [41, 43, 46, 51]. Studies in the US have shown interest amongst adolescents and young people in LAART [17, 52].

However, there is currently very limited evidence on the preference for LAART among AYLHIV in sub-Saharan Africa, and factors associated with these preferences, with only one recent qualitative paper published which included 16 young people (under age 25) from Kenya [53]. The study found mostly positive initial reactions to LAART, with the main advantages identified were relieving the daily pill-burden and improved confidentiality, preventing unintentional HIV status disclosure, as well as helping to improve adherence [53].

This study analyses 2017–2018 data from a large study of AYLHIV in South Africa to understand which AYLHIV are more likely to access and benefit from the rollout of LAART in a resource-constrained setting, such as a health district in the Eastern Cape Province, South Africa. We investigate socio-demographic, HIV and medication-related factors. Finally, based on findings documenting the differences by sex in accessing biomedical and behavioural interventions among adolescent boys and girls in this setting, we explore the effect of AYLHIV sex on LAART preferences and the factors associated with these preferences [54, 55].

## Methods

We conducted a cross-sectional analysis of factors associated with LAART preferences using third round data from a three-wave Mzantsi Wakho cohort main study, given that the outcome measure—LAART preference—was only collected in the third round when LAART efficacy data became available [31]. This is a mixed-methods cohort study of 1176 HIV-positive adolescents (10–19 years old at baseline in 2014–2015) recruited from the Eastern Cape Province of South Africa. Ethical approval for this study was provided by Research Ethics Committees at the Universities of Oxford (SSD/CUREC2/12-21) and Cape Town (CSSR 2013/4; 2019/1), Eastern Cape Departments of Health and Basic Education, and participating health facilities.

### Participants and Procedures

We identified 53 health facilities (primary health clinics, community healthcare centres, and hospitals) providing HIV care to adolescents in the Buffalo City District in the Eastern Cape Province. This area was selected in consultation with local government partners as a historically disadvantaged region with poor infrastructure and high HIV-prevalence rates [56]. In each facility, all clinic patient files were reviewed to identify adolescents who had initiated HIV treatment and were aged 10–19 years. 1176 adolescents living with HIV were identified and recruited in clinics, or traced back into their home communities, to ensure the inclusion of those who were no longer engaged in care. All adolescents and their caregivers (when adolescents were under 18) participating in the study gave voluntary, written informed consent following international and national guidelines for consent among vulnerable populations. In cases of limited literacy, the consent was read aloud to the participants. The study did not provide any financial incentives to participants. Based on the recommendation of the study’s adolescent advisory group, all adolescents received a small gift pack (including pencils, deodorant, and soap), a snack, and a certificate of participation at each study visit regardless of whether or not they participated in the study. In order to avoid unwanted disclosure or stigma, all study materials (including the certificate) did not include any references to HIV/AIDS, but focused on overall health and well-being of young people, including those taking long-term medication.

Data for this analyses was collected using quantitative questionnaires developed with input from a adolescent advisory group, translated into local language (Xhosa) and back-translated for improved conceptual validity [57], then piloted with 25 adolescents at baseline. They were designed to be non-stigmatising and engaging by including graphics, interactive games and vignettes to introduce questions around sensitive topics. The questionnaires were adapted at each successive wave based on prior data collection wave with feedback from participants and the study team. The questionnaire was embedded onto a tablet which was given to each participant for completion. Before the interview, trained community-based research assistants sat with the adolescents to demonstrate how to use the tablet properly and guide them when necessary. The participants then completed the questionnaire on their own in either English or Xhosa depending on their preference and lasted between 60 to 90 min. In the third wave of the study, additional questions were included that reflected the older age of the cohort (12–24 years during the third interview in 2017–2018). Individual questionnaires were conducted in clinics or communities by researchers trained in working with vulnerable adolescents.

**Fig. 1:**
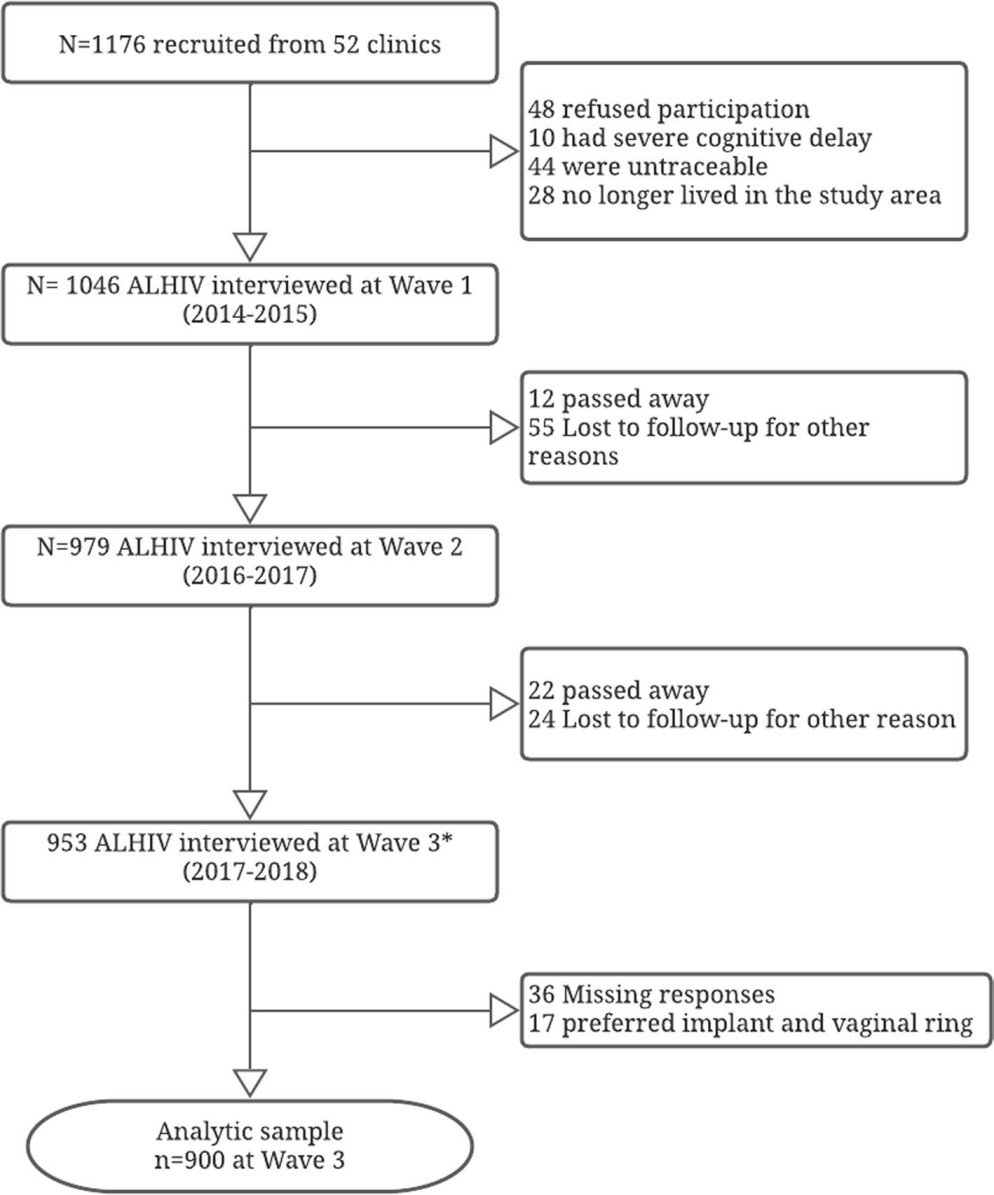
Study sample flowchart. *933 participants were interviewed across all the data collection waves and 20 participants included here were those interviewed at baseline and at Wave 3 but not Wave 2

Of all study-eligible adolescents 1046 (90.1%) were recruited (55% female) and participated at baseline of the study in 2014–2015, 4.1% refused participation (either adolescent or caregiver), 0.9% had very severe cognitive delay, 3.7% were untraceable, and 1.2% no longer lived in the area. There were no statistically significant differences on age, sex, and rural/urban residence between adolescents who were and were not recruited [21]. All adolescents who had given consent to be re-approached were invited to participate in the 2016–2017 and 2017–2018 rounds of data collection. Out of 1046 who participated at baseline, 94% were followed up at wave 2 of the study, and 91% at wave 3. Among reasons for attrition, some participants could not be traced, or were not willing or available to participate, and 34 (3.3%) adolescents died during the study period. Our analyses focus on the 953 participants who participated at baseline and were retained in the third and final wave of the study (see Fig. 1 for the recruitment flow diagram).

### Measures

Full questionnaires are available at http://www.mzantsiwakho.co.za/. The primary outcome for analysis reported in this manuscript is LAART preference based on responses to the question “Researchers are developing many different types of medicines. If you had a choice, how would you prefer to take your ARVs/ HIV medicine?” Possible response options included (1) Pills-several or- one pill a day, (2) Injection-monthly or every three months, (3) Implants, and (4) Vaginal ring. Participants who reported preferring injectable every one or three months were coded as ‘1’, and ‘0’ for pill-based regimens. Participants reporting preferences for implant or vaginal ring delivery (n = 17) were excluded from these analyses due to the small size of this subgroup. The primary outcome was assessed at the third wave of data collection, with the item added in response to the availability of LAART efficacy evidence.

*Socio-demographic items* including *age, sex, urban/rural residence, type of housing*, and *orphanhood*. *Food insecurity* was measured based on not affording meals the entire week and 3 meals per day, using national survey measures [58]. *Household poverty* was measured as access to the eight highest socially-perceived necessities for children and adolescents validated in a nationally-representative South African Social Survey (e.g., enough food and can afford a doctor). *School progression* was measured as ‘1’ if the adolescent did not repeat last grade and’0’ otherwise.

*HIV-status related variables* included: *Mode of HIV acquisition* (recently, i.e. sexually, versus vertically acquired HIV) was estimated using age of ART initiation cut-off (≤ 10 years), following existing sub-Saharan African paediatric cohorts [59, 60], validated with other strong evidence (i.e. self-reported sexual history and parental death) in the absence of medical record on mode of HIV acquisition [61]. *Recent ART initiation* was defined as being on antiretroviral therapy for 2 years or less. *Recent knowledge of HIV status* was defined as learning one’s HIV status in the past 2 years*. Experiences of HIV-related stigma* was measured as a dichotomy based on experiencing any of the HIV-related stigma items from the ALHIV Stigma Scale (ALHIV-SS) developed in collaboration with ALHIV in South Africa and shown to have strong psychometric properties [62].

*Medication-related factors* included experience of any ART medication related *side-effects,* whether their *treatment changed in the past year, self-reported pill burden,* and if they are taking *multiple medications* defined as taking any other medication, including injections or family planning-related medications. We included two methods of contraception use by female participants or partners of male participants as dichotomous variables, namely, injection and implant, to investigate whether exposure to these modalities shaped LAART preferences.

We also included *HIV care and treatment service variables* namely: whether they were *retained in care*, defined as self-reported adherence and clinic engagement, following WHO guidelines [50]. *Self-reported adherence* was measured as a binary indicator of missed doses in the past seven days adherence (including weekdays and weekends), based on currently taking ART and not having missed any doses in the past 7 days [63]. This measure was assessed using adapted items from the Patient Medication Adherence Questionnaire and measures developed in Botswana [64]. *Clinic engagement* was measured as consistent attendance of scheduled clinic appointments in the last year. Additional HIV care and treatment service variables included: having a *treatment buddy* and being part of a *support group*, and if their medicine was *stocked out* at health facilities in the last year.

### Analyses

Analyses was conducted in STATA.v16 and followed three steps. Firstly, we compared socio-demographic factors for complete observations and those lost to follow-up (LFTU) in this cohort using Chi-square test statistic. Secondly, adolescents who reported preferring monthly injections or injection every 3 months at the third and final wave were compared to all other adolescents across all factors in bivariate analyses. Thirdly, stepwise multivariate regression models were run with injectable preference as an outcome including all individual, HIV, and medication-taking predictors, controlling for socio-demographic characteristics. Following Hosmer and Lemeshow [65], in the first model, all potential predictors and covariates were included. In the second model, only factors significant at the 10% level (p < 0.10) were retained. In the third model, only factors significant at (p < 0.05) were entered, to maximize analyses power while taking potential covariates into account. For the final model, p-values < 0.05 were considered to be statistically significant. Next, predicted probabilities of preferring LAART were computed using marginal effects modelling for factors significantly associated with the outcome in the third model in the prior step. Fourth, we modelled differences by sex of factors significantly associated with LAART preferences, by testing the interaction of sex with each factor, adjusting for testing multiple moderators concurrently.

## Results

### Socio-demographic Characteristics

Nine hundred and fifty-three participants interviewed at baseline were re-interviewed at the third and final wave of this study. Of these, 17 participants preferred implant and vaginal ring and 36 (3.8%) had a missing response for LAART preference due to the question being asked only to participants who disclosed their HIV status to the research team during the interview hence, they were excluded from the analysis. When compared to those included, participants excluded from the analysis were more likely to be female (χ^2^ = 7.7, p = 0.005), and to have recently acquired HIV (χ^2^ = 6.0, p = 0.014). An assessment of the total cohort sample retained versus those lost-to-study follow-up participants showed no significant differences except that those lost-to-study follow-up were more likely to be older (χ^2^ = 8.8, p = 0.003) (Additional file 1: Supplementary Table).

Nearly one in eight (n = 111/953, 11.6%) of sample reported preference for LAART over single or multiple pill regimens. As shown in Table 1, included participants (N = 900) were 54.1% female, about a quarter lived in rural communities, and 14% lived in informal housing, a shack in its own plot or a backyard. Most of these participants resided in resource-constrained settings: over two thirds lived in poor households, a quarter were double orphans, nearly half experienced past-week food insecurity, and just over half did not repeat any grades in school. Over three-quarters acquired HIV vertically, a quarter found out about their HIV-positive status recently, and one in ten initiated ART in the last two years. In bivariate analyses (Table 1), participants who preferred LAART were more likely to report: past-week food insecurity (χ^2^ = 9.2, p = 0.002), have recently initiated on ART (χ^2^ = 4.5, p = 0.033), experiencing greater levels of ART side effects (χ^2^ = 14.7, p < 0.001), a higher pill burden (χ^2^ = 14.9, p < 0.001), any HIV-related stigma (χ^2^ = 14.5, p < 0.001), experiencing stock outs in the past year (χ^2^ = 12.6, p < 0.001), and less likely to be retained in care (χ^2^ = 8.7, p = 0.003).

**Table 1:** Bivariate associations between LAART preferences and hypothesised factors

	Long-acting injectable ART (LAART) preference					
**Factor group**	**Factor** **N (%)**	**Total** **(N = 900)**	**LAART** **preferred** **(n = 111)**	**Oral ART preference** **(n = 789)**	**Chi-square (**χ^2^) **Statistic**	**p-value**
**Socio-demographic**	Age (15 + years)	601 (66.8)	78 (70.3)	523 (66.3)	0.7	0.404
	Female	487 (54.1)	54 (48.7)	433 (54.9)	1.5	0.217
	Rural residence	214 (23.8)	29 (24.3)	187 (23.8)	0.02	0.896
	Informal housing	127 (14.1)	14 (12.6)	113 (14.3)	0.2	0.625
	Double orphan	225 (25.0)	33 (29.7)	192 (24.3)	1.5	0.219
	Food insecurity	422 (46.9)	67 (60.4)	355 (45.0)	9.2	0.002*
	Poverty	605 (67.2)	78 (70.3)	527 (66.8)	0.5	0.465
	School progression	526 (58.4)	70 (63.1)	456 (57.8)	1.1	0.292
**HIV-status related variables**	Vertically acquired HIV	711 (79.2)	90 (81.1)	621 (78.9)	0.3	0.597
	Recent ART initiation	81 (9.0)	16 (14.4)	65 (8.2)	4.5	0.033*
	Recently found about HIV status	192 (21.5)	26 (23.4)	166 (21.2)	0.3	0.589
	HIV stigma	153 (17.0)	33 (29.7)	120 (15.2)	14.5	< 0.001*
**Medication-related factors**	Medication side-effects	160 (17.8)	34 (30.9)	126 (16.0)	14.7	< 0.001*
	Past year treatment changes	386 (42.9)	55 (49.6)	331 (42.0)	2.3	0.130
	Pill burden	198 (22.1)	40 (36.4)		14.9	< 0.001*
	Multiple medication types	365 (40.6)	54 (48.7)	311 (39.4)	3.4	0.064
	Contraception (injection)	135 (15.0)	14 (12.6)	121 (15.3)	0.6	0.452
	Contraception (implant)	18 (2.0)	3 (2.7)	15 (1.9)	0.3	0.572
**HIV care and treatment service variables**	Retention in care	656 (72.9)	68 (61.3)	588 (74.5)	8.7	0.003*
	Treatment buddy	536 (59.6)	57 (51.4)	479 (60.7)	3.5	0.060
	Support group attendance	67 (7.4)	12 (10.8)	55 (7.0)	2.1	0.149
	Stock-outs	81 (9.0)	20 (18.0)	61 (7.7)	12.6	< 0.001*

*p-value of ≤ 0.05 was considered statistically significant

### Factors Associated with LAART Preferences in Multivariate Analyses

In multivariate analyses, six factors were associated with preference for LAART (Table 2., Model 3). Recently initiated adolescents were more than twice as likely to prefer LAART (aOR = 2.02, 95% CI 1.09–3.74, p = 0.025), and those who experienced any HIV-related stigma were more than two times as likely to report LAART preference (aOR 2.22, 95% CI 1.39–3.53, p = 0.001). Three medication-related factors were associated with preference for LAART over pill-based regimens: side effects, treatment changes, and pill burden. Participants who experienced side-effects taking their current regimens—all pill-based—were 84% more likely to prefer LAART (aOR = 1.84, 95% CI 1.15–2.97, p = 0.012). Participants who had experienced medication changes in the last year were 63% more likely to prefer LAART (aOR = 1.63, 95% CI 1.06–2.51, p = 0.025), while those who had to take multiple pills were 88% more likely to prefer LAART (aOR = 1.88, 95% CI 1.20–2.94, p = 0.006). Participants who received care in facilities that experienced stock outs in the last year were more than twice as likely to report LAART preferences (aOR = 2.56, 95% CI 1.40–4.68, p = 0.002).

**Table 2.** Multivariate regression results for LAART preferences of adolescents and young people living with HIV

Factors	Long-acting injectable ART (LAART) preference					
	Model 1 (Full Model) N = 889		Model 2 (p < 0.10) N = 900		Model 3 (p < 0.05) N = 900	
	ORs (95% CI)	p-value	ORs (95% CI)	p-value	ORs (95% CI)	p-value
Socio-demographic						
Age (15 + years)	1.17 (0.70–1.96)	0.544				
Female	0.67 (0.43–1.05)	0.081	0.67 (0.44–1.03)	0.067		
Rural residence	1.19 (0.71–1.98)	0.517				
Informal housing	0.91 (0.48–1.71)	0.771				
Double orphan	1.16 (0.71–1.89)	0.552				
Food Insecurity	1.59 (1.02–2.49)	0.039	1.52 (0.99–2.33)	0.056		
Poverty	1.10 (0.69–1.75)	0.698				
School progression	1.30 (0.83–2.04)	0.245				
HIV-status related variables						
Vertically acquired HIV	1.25 (0.68–2.29)	0.478				
Recent ART initiation	2.14 (1.03–4.45)	0.043	2.02 (1.08–3.78)	0.027	2.02 (1.09–3.74)	0.025*
Recently found about HIV status	1.08 (0.60–1.94)	0.795				
HIV stigma	2.36 (1.45–3.83)	0.001	2.26 (1.41–3.62)	0.001	2.22 (1.39–3.53)	0.001*
Medication-related factors						
Medication side-effects	1.77 (1.06–2.95)	0.030	1.87 (1.15–3.06)	0.012	1.84 (1.15–2.97)	0.012*
Past year treatment changes	1.47 (0.94–2.29)	0.092	1.56 (1.01–2.41)	0.043	1.63 (1.06–2.51)	0.025*
Pill burden	1.63 (1.01–2.63)	0.045	1.74 (1.10–2.75)	0.017	1.88 (1.20–2.94)	0.006*
Multiple medication types	1.45 (0.89–2.36)	0.132				
Contraception (injection)	0.66 (0.32–1.37)	0.265				
Contraception (implant)	1.38 (0.36–5.31)	0.635				
HIV care and treatment service variables						
Retention in care	1.10 (0.66–1.86)	0.708				
Treatment buddy	0.78 (0.50–1.22)	0.275				
Support group attendance	1.56 (0.77–3.18)	0.217				
Stock-outs	2.45 (1.26–4.75)	0.008	2.48 (1.34–4.57)	0.004	2.56 (1.40–4.68)	0.002*

^*^p-value of ≤ 0.05 was considered statistically significant

Based on our multivariate regression model, to ease interpretation we estimated the marginal probabilities for each of the six factors or their combination. Assuming that the distribution of all the factors remained the same among adolescents, and adolescents did not experience any of the factors, 6% would be likely to prefer LAART. Each factor alone resulted in the probability of reporting LAART preferences increasing to between 9 and 13%. If adolescents experienced all the factors combined (HIV-related stigma, side-effects, treatment change, pill burden, and stock outs), over two thirds (66%) would report LAART preference.

### LAART Preferences by Sex

**Fig. 2:**
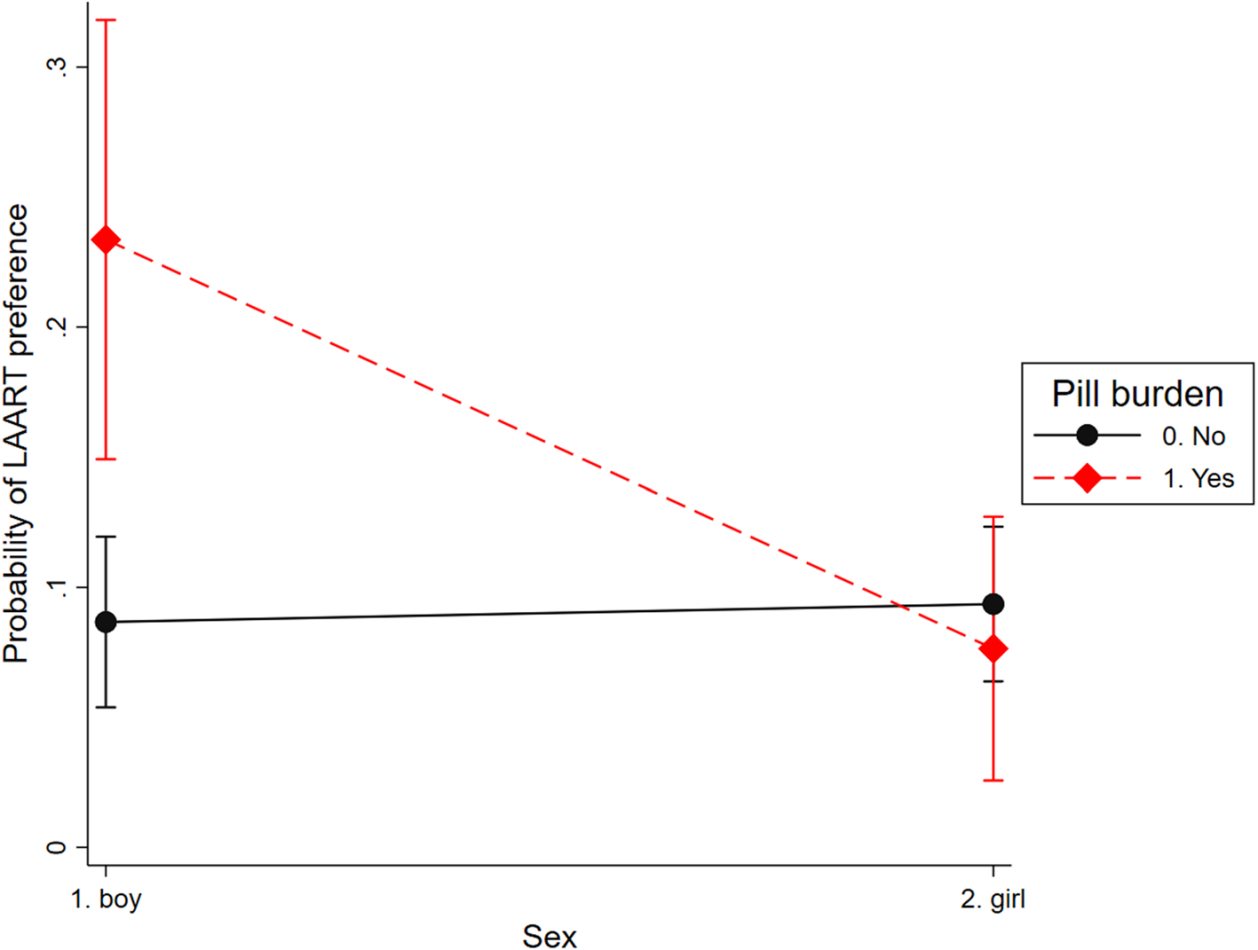
A moderator analysis between pill burden and LAART preference

Preferences for LAART did not differ by sex in univariate and multivariate analyses, however we based on qualitative data from South Africa and similar settings, we tested whether the associations between the factors in multivariate analysis and LAART preference differed by sex. Moderation by participant sex (female/ male) results (Table 3.) suggest that the effect of pill burden on LAART preference varies by participant sex: adolescent boys living with HIV who reported pill burden were more likely to prefer LAART than adolescent girls (Fig. 2).

**Table 3:** Sex moderation for LAART injectable preferences among AYLHIV

	LAART preferences (N = 900)	
**Factors**	**OR 95%CI**	**p-value**
Female	0.79 (0.35–1.79)	0.569
Recent ART initiation	1.33 (0.41–4.29)	0.631
Side-effects	2.0 (0.95–4.20)	0.067
Past-year treatment changes	1.85 (1.01–3.40)	0.048*
Pill burden	3.28 (1.78–6.04)	< 0.001*
Stock-outs	1.18 (0.39–3.51)	0.772
HIV stigma	1.60 (0.76–3.37)	0.213
Female * Recent ART initiation	2.01 (0.50–8.18)	0.328
Female * Side-effects	1.02 (0.38–2.74)	0.967
Female * Past-year treatment changes	0.80 (0.33–1.92)	0.614
Female * Pill burden	0.27 (0.10–0.71)	0.008*
Female * Stock-outs	3.50 (0.92–13.3)	0.066
Female * HIV stigma	1.70 (0.64–4.52)	0.291

*p-value of ≤ 0.05 was considered statistically significant

## Discussion

Recently, Phase 3 clinical trials have shown LAART to be a promising alternative and non-inferior to daily oral ART [28–31] and the first LAART was recently approved by the National Institute for Health and Care Excellence (NICE) in the UK [66]. However, little data is available on LAART preferences among adolescents and young people already living with HIV who are critical to breaking the HIV transmission cycle and reaching the UNAIDS 95–95–95 targets. We document factors associated with preferences for LAART among AYLHIV initiated on ART in 53 public health facilities in the Eastern Cape province of South Africa. HIV-related stigma and several medication-related factors were associated with LAART preferences in this large South African sample of adolescents and young people living with HIV.

Only one in eight participants preferred LAART compared to single or multiple-pill regimens. Although the study team explained the concept of LAART to participants, lack of exposure and limited knowledge of additional delivery modes may have shaped their responses. This low prevalence for LAART preference may also be due to the timing of data collection, which was in 2017–2018, before LAART were available through public health systems globally, with no information or availability in the Eastern Cape province. Study participants had some exposure to injectable medications such as contraception, but very limited exposure to injectable PrEP, which may have improved understanding of LAART. In-depth qualitative work should be conducted to understand interest, preferences and additional barriers to accessing injectable rather than pill-based medication. We would expect that exposure to LAART information and stock would shift these preferences. Nonetheless, our findings provide important early evidence on the possible impact of expanding HIV treatment options for this vulnerable age-group. They suggest that increasing the range of available treatment options (pills, implants, injections, rings) may address some of the barriers they experience in attaining and sustaining viral suppression.

Stigma was associated with preference for LAART. This finding resonates with prior analyses highlighting the negative impact of stigma on HIV-related outcomes, including retention in care [67]. Several medication-related factors (medication side-effects, treatment changes, and pill burden) were associated with higher preferences for LAART. Each of these factors is also individually associated with reduced ART non-adherence [2, 68, 69], and thus poorer rates of viral suppression [68, 70]. The differential preference for LAART among adolescent boys who reported experiencing ART pill burden suggests that LAART may also support adolescent boys and young men living with HIV attain and sustain good HIV outcomes, including higher HIV testing and treatment initiation rates, where adolescent boys and young men fall behind [71]. Further, ART stockouts were associated with higher preferences for LAART and have been strongly associated with poor retention in care among AYLHIV in this cohort [[21]] and other age groups [69]. Monthly or quarterly (every three months) LAART would enable adolescents to have greater agency over their clinic visits and medication-taking practices. Multi-month prescribing has been recommended by the WHO for clinically stable people living with HIV to decrease the burden of care since 2016 [72]. Refills every 6 months were more widely implemented as a priority for clinically stable HIV patients in various sub-Saharan countries including South Africa due to the COVID-19 pandemic to limit clinic visits [73–75]. However, there is no data on how AYLHIV are accessing multi-month prescriptions during the COVID-19 pandemic in resource-constrained settings. Less frequent visits and medication dispensing needs would also reduce the negative impact of stockouts on poor ART outcomes and retention in care documented in this analyses and baseline data from this cohort [21].

Finally, LAART is a promising tool to operationalize one of the approaches that AYLHIV request as part of differentiated service delivery: less frequent clinic visits and medication refills [76]. By offering LAART to AYLHIV who are currently struggling to take their ART due to side-effects, medication changes, pill burden or stockouts, we can support AYLHIV meet the third 95. In particular, LAART may be a key factor in facilitating adherence and viral suppression among adolescent boys and young men, who have poorer HIV outcomes than their female peers. Moreover, in the context of over-stretched healthcare service provision during COVID-19, adolescents and young people have noted an increase in stigma related to attending clinics, due to increased visibility to respect social distancing requirements. Less frequent visits may attenuate the negative effect of stigma and other barriers to access to and retention in HIV care among AYLHIV [77].

This study has several limitations. Firstly, the data presented here is cross-sectional as the outcome was only measured in the final wave of cohort data collection. Secondly, AYLHIV expressed hypothetical preferences on how they would like to take their ART without testing or trying out actual alternative treatment or mode of delivery such as injectables, implants or vaginal rings (among female participants). Thirdly, we did not measure hesitancy towards injections. Although this study did not have such data available, existing research has documented potential barriers to LAART and injectable PrEP [51, 78]. These included worries about side effects and pain which came with injections—especially intramuscular ones such as LAART, increased visits to the clinic, safety fears, and abandoning a regime that they know works and are used to [51, 78]. However, these were all perceived by adult female participants to be more manageable than the logistical and psychological burdens and other concerns related to taking daily oral ART, especially in a low resource context [33]. Furthermore, there is also a history of mistrusting public health practices and biomedical products combined with HIV-related conspiracy beliefs in South Africa, including a mistrust towards needles and injections with the belief that they can harm [79–81]. This is also an issue that has arisen due to the development of the COVID-19 vaccine [82]. Recent qualitative research with adolescent research advisors in South Africa, suggests that trust in service provision may also influence the uptake of new biomedical products, such as injectable medication or vaccines [83]. Fourth, the data was collected in South Africa and may not be generalizable to other settings. Finally, we were not able to include in our analysis prior exposure to non-pill-based medication delivery modes such as injections, implants and insertive health products (such as intrauterine devices, IUDs). Although this exposure may explain some of the preferences—or lack thereof—for LAART documented in this study, rates of implants and IUDs among female AYLHIV were too small to allow analyses. Additional analyses by sex should investigate the role of injectable hormonal contraception use history on LAART preferences, especially given recent products that combine contraception and ART.

Our study’s findings have important implications for research and development of LAART and for delivering differentiated services for AYLHIV. While not all AYLHIV will choose LAART, even when they are aware of it and have been able to test it, having the option to offer ART less frequently and through non-pill modalities may ensure that we better reach the adolescents and young people with unique needs that are unmet by current ART delivery modalities. However in order to successfully implement LAART in low-resource settings, there will need to be strong collaborative efforts between international agencies, the pharmaceutical industry and the manufacturing sector [84]. The logistical requirements and challenges with governmental and health systems will need to be carefully navigated in order to ensure the effective introduction of LAART [85]. Moreover, trials that test LAART awareness, acceptability, and models for large-scale accessibility, particularly among younger adolescents will be critical to extend the time on first line treatment among younger adolescents.

## Conclusions

Adolescents and young people living with HIV have poorer HIV treatment outcomes than all other age groups, necessitating a broad range of support options to achieve and sustain viral suppression. Findings from this study add to a burgeoning body of evidence on the importance of offering a toolkit of interventions to ensure that they survive and thrive and meet global targets for HIV prevention and treatment. We suggest that LAART is one such intervention with the potential to support adolescents experiencing specific treatment-related challenges that are currently unaddressed. Progress in injectable PrEP, in particular since the first injectable was recently approved by the U.S. Food and Drug Administration [86–88], offers an opportunity to developing, testing and making this treatment modality available to AYLHIV. In particular, future trials which test LAART regimens and delivery modalities must include adolescents to document the regimen’s efficacy, safety and acceptability for this age group given the rates of LAART preferences documented in this study (only 12%). Moreover, mixed methods research is needed to further explore the acceptability of different modalities and their scalability. Expanding ART delivery options to include LAART could improve adherence outcomes and reduce the burden on healthcare systems. This is especially important for populations with persistent and dismal health outcomes—such as adolescents living with HIV—to meet the 3rd 95 and thrive into adulthood.

## Supplementary Information

Below is the link to the electronic supplementary material.

Supplementary file1 (DOCX 15 KB)

## Data Availability

Data is available upon request following correct procedures (for more information please see: https://www.mzantsiwakho.org.za/publications). Data is not yet available open access as the study has not concluded, therefore data is not fully anonymised.

## Abbreviations

AIDS: Acquired immunodeficiency syndrome
AYLHIV: Adolescent and youth living with HIV
HIV: Human immunodeficiency virus
LAART: Long-acting injectable antiretroviral therapy

## Code Availability

STATA do-file available upon request from co-authors.

## References

[1] UNAIDS JUNP on H. At a Glance - HIV among Women and Girls in Sub-Saharan Africa. 2019.

[2] Reif LK, Abrams EJ, Arpadi S, Elul B, McNairy ML, Fitzgerald DW (2020). Interventions to improve antiretroviral therapy adherence among adolescents and youth in low- and middle-income countries: a systematic review 2015–2019. AIDS Behav.

[3] UNICEF. Adolescent HIV prevention - UNICEF DATA. https://data.unicef.org/topic/hivaids/adolescents-young-people/. 2020. accessed 25 July 2020.

[4] UNAIDS. Understanding Fast-Track: accelerating action to end the AIDS epidemic by 2030. https://www.unaids.org/sites/default/files/media_asset/201506_JC2743_Understanding_FastTrack_en.pdf. 2015. accessed 3 May 2022.

[5] UNAIDS. Ending the AIDS epidemic for adolescents, with adolescents: A practical guide to meaningfully engage adolescents in the AIDS response. https://www.unaids.org/sites/default/files/media_asset/ending-AIDS-epidemic-adolescents_en.pdf. 2016. accessed 3 May 2022.

[6] Nachega JB, Mills EJ, Schechter M (2010). Antiretroviral therapy adherence and retention in care in middle-income and low-income countries: current status of knowledge and research priorities. Curr Opin HIV AIDS.

[7] Lamb MR, Fayorsey R, Nuwagaba-Biribonwoha H, Viola V, Mutabazi V, Alwar T (2014). High attrition before and after ART initiation among youth (15–24 years of age) enrolled in HIV care. AIDS.

[8] Adejumo OA, Malee KM, Ryscavage P, Hunter SJ, Taiwo BO (2015). Contemporary issues on the epidemiology and antiretroviral adherence of HIV-infected adolescents in sub-Saharan Africa: a narrative review. J Int AIDS Soc.

[9] Salou M, Dagnra AY, Butel C, Vidal N, Serrano L, Takassi E (2016). High rates of virological failure and drug resistance in perinatally HIV-1-infected children and adolescents receiving lifelong antiretroviral therapy in routine clinics in Togo. J Int AIDS Soc.

[10] Ryscavage PA, Anderson EJ, Sutton SH, Reddy S, Taiwo B (2011). Clinical outcomes of adolescents and young adults in adult HIV care. J Acquir Immune Defic Syndr.

[11] Ferrand RA, Briggs D, Ferguson J, Penazzato M, Armstrong A, Macpherson P (2016). Viral suppression in adolescents on antiretroviral treatment: review of the literature and critical appraisal of methodological challenges. Trop Med Int Health.

[12] Mukui IN, NgAngA L, Williamson J, Wamicwe JN, Vakil S, Katana A (2016). Rates and predictors of non-adherence to antiretroviral therapy among HIV-positive individuals in Kenya: results from the second Kenya AIDS indicator survey, 2012. PLoS ONE.

[13] Cherutich P, Kim AA, Kellogg TA, Sherr K, Waruru A, De Cock KM (2016). Detectable HIV viral load in Kenya: data from a population-based survey. PLoS ONE.

[14] Zhou S, Cluver L, Shenderovich Y, Toska E (2021). Uncovering ART adherence inconsistencies: an assessment of sustained adherence among adolescents in South Africa. J Int AIDS Soc.

[15] Volberding PA, Deeks SG (2020). Antiretroviral therapy and management of HIV infection. Lancet.

[16] Lorenzo-Redondo R, Fryer HR, Bedford T, Kim EY, Archer J, Kosakovsky Pond SL (2016). Persistent HIV-1 replication maintains the tissue reservoir during therapy. Nature.

[17] Nachman S, Townsend CL, Abrams EJ, Archary M, Capparelli E, Clayden P (2019). Long-acting or extended-release antiretroviral products for HIV treatment and prevention in infants, children, adolescents, and pregnant and breastfeeding women: knowledge gaps and research priorities. Lancet HIV.

[18] WHO. WHO | HIV and adolescents: Guidance for HIV testing and counselling and care for adolescents living with HIV. World Health Organization. http://www.who.int/hiv/pub/guidelines/adolescents/en/ 2017. Accessed 24 July 2020

[19] Pettifor A, Stoner M, Pike C, Bekker LG (2018). Adolescent lives matter: preventing HIV in adolescents. Current Opin HIV AIDS.

[20] Cluver LD, Toska E, Orkin FM, Meinck F, Hodes R, Yakubovich AR (2016). Achieving equity in HIV-treatment outcomes: can social protection improve adolescent ART-adherence in South Africa?. AIDS Care.

[21] Cluver L, Pantelic M, Toska E, Orkin M, Casale M, Bungane N (2018). STACKing the odds for adolescent survival: health service factors associated with full retention in care and adherence amongst adolescents living with HIV in South Africa. J Int AIDS Soc.

[22] UNICEF. For Every Child, End AIDS: Seventh Stocktaking Report, 2016. https://www.unicef.org/publications/index_93427.html 2016. accessed 24 July 2020.

[23] Kim SH, Gerver SM, Fidler S, Ward H (2014). Adherence to antiretroviral therapy in adolescents living with HIV: systematic review and meta-analysis. AIDS.

[24] Toska E, Zhou S, Laurenzi CA, Haghighat R, Saal W, Gulaid L (2022). Predictors of secondary HIV transmission risk in a cohort of adolescents living with HIV in South Africa. AIDS.

[25] Joint United Nations Programme on HIV/AIDS (UNAIDS). Fast-Track strategy to end the AIDS epidemic by 2030 | UNAIDS. https://www.unaids.org/en/resources/campaigns/World-AIDS-Day-Report-2014. accessed 24 July 2020.

[26] Casale M, Carlqvist A, Cluver L (2019). Recent interventions to improve retention in HIV care and adherence to antiretroviral treatment among adolescents and youth: a systematic review. AIDS Patient Care STDS.

[27] Ridgeway K, Dulli LS, Murray KR, Silverstein H, Dal Santo L, Olsen P (2018). Interventions to improve antiretroviral therapy adherence among adolescents in low- and middle-income countries: a systematic review of the literature. PLoS ONE.

[28] Andrews CD, Heneine W (2015). Cabotegravir long-acting for HIV-1 prevention. Current Opin HIV AIDS.

[29] Damico R, Margolis DA (2020). Long-acting injectable therapy: an emerging paradigm for the treatment of HIV infection. Current Opin HIV AIDS.

[30] Spreen WR, Margolis DA, Pottage JC (2013). Long-acting injectable antiretrovirals for HIV treatment and prevention. Current Opin HIV AIDS..

[31] Margolis DA, Gonzalez-Garcia J, Stellbrink HJ, Eron JJ, Yazdanpanah Y, Podzamczer D (2017). Long-acting intramuscular cabotegravir and rilpivirine in adults with HIV-1 infection (LATTE-2): 96-week results of a randomised, open-label, phase 2b, non-inferiority trial. Lancet.

[32] Bares SH, Scarsi KK (2022). A new paradigm for antiretroviral delivery: Long-acting cabotegravir and rilpivirine for the treatment and prevention of HIV. Curr Opin HIV AIDS.

[33] Kerrigan D, Sanchez Karver T, Muraleetharan O, Savage V, Mbwambo J, Donastorg Y (2020). “A dream come true”: perspectives on long-acting injectable antiretroviral therapy among female sex workers living with HIV from the Dominican Republic and Tanzania. PLoS ONE.

[34] Weiser SD, Anema A, Vogenthaler N, Frongillo EA, Kadiyala S (2009). Food insecurity and HIV/AIDS: current knowledge, gaps, and research priorities. Current HIV/AIDS Rep.

[35] Singer AW, Weiser SD, McCoy SI (2015). Does food insecurity undermine adherence to antiretroviral therapy? A systematic review. AIDS Behav.

[36] Elion R. Updates on the use of long-acting ART in HIV prevention programs. Opportunities, and limitations of these new drug modalities. Academic Medical Education Online Meeting Highlights 2020. 2020.

[37] Gendelman HE, McMillan JE, Bade AN, Edagwa B, Kevadiya BD (2019). The promise of long-acting antiretroviral therapies: from need to manufacture. Trends Microbiol.

[38] Weld ED, Flexner C (2020). Long-acting implants to treat and prevent HIV infection. Current Opin HIV AIDS.

[39] Castor D, Meyers K, Allen S (2020). The only way is up: priorities for implementing long-acting antiretrovirals for HIV prevention and treatment. Current Opin HIV AIDS..

[40] Culhane J, Sharma M, Wilson K, Roberts DAA, Mugo C, Wamalwa D (2020). Modeling the health impact and cost threshold of long-acting ART for adolescents and young adults in Kenya. EClinicalMedicine..

[41] Margolis DA, Boffito M (2015). Long-acting antiviral agents for HIV treatment. Current Opin HIV AIDS.

[42] Rusconi S, Marcotullio S, Cingolani A (2017). Long-acting agents for HIV infection: biological aspects, role in treatment and prevention, and patient’s perspective. New Microbiol.

[43] Kerrigan D, Mantsios A, Gorgolas M, Montes ML, Pulido F, Brinson C (2018). Experiences with long acting injectable ART: A qualitative study among PLHIV participating in a Phase II study of cabotegravir + rilpivirine (LATTE-2) in the United States and Spain. PLoS ONE.

[44] Fernandez C, Halsema van CL.  (2019). Evaluating cabotegravir/rilpivirine long-acting, injectable in the treatment of HIV infection: emerging data and therapeutic potential. HIV/AIDS Res Palliative Care..

[45] Murray M, Pulido F, Mills A, Ramgopal M, LeBlanc R, Jaeger H (2019). Patient-reported tolerability and acceptability of cabotegravir + rilpivirine long-acting injections for the treatment of HIV-1 infection: 96-week results from the randomized LATTE-2 study. HIV Res Clin Pract..

[46] Simoni JM, Beima-Sofie K, Mohamed ZH, Christodoulou J, Tapia K, Graham SM (2019). Long-acting injectable antiretroviral treatment acceptability and preferences: a qualitative study among US providers, adults living with HIV, and parents of youth living with HIV. AIDS Patient Care STDS.

[47] Simoni JM, Tapia K, Lee SJ, Graham SM, Beima-Sofie K, Mohamed ZH (2020). A conjoint analysis of the acceptability of targeted long-acting injectable antiretroviral therapy among persons living with HIV in the US. AIDS Behav.

[48] Dandachi D, Dang BN, Lucari B, Swindells S, Giordano TP (2020). Acceptability and preferences for long-acting antiretroviral formulations among people with HIV infection. AIDS Care.

[49] Mantsios A, Murray M, Karver TS, Davis W, Margolis D, Kumar P (2020). Efficacy and freedom: patient experiences with the transition from daily oral to long-acting injectable antiretroviral therapy to treat HIV in the context of phase 3 trials. AIDS Behav.

[50] Carillon S, Gallardo L, Linard F, Chakvetadze C, Viard JP, Cros A (2020). Perspectives of injectable long acting antiretroviral therapies for HIV treatment or prevention: understanding potential users’ ambivalences. AIDS Care.

[51] Philbin MM, Parish C, Kinnard EN, Reed SE, Kerrigan D, Alcaide M (2020). A multi-site study of women living with HIV’s perceived barriers to, and interest in, long-acting injectable anti-retroviral therapy. JAIDS J Acquir Immune Defic Syndr.

[52] Weld ED, Rana MS, Dallas RH, Camacho-Gonzalez AF, Ryscavage P, Gaur AH (2019). Interest of youth living with HIV in long-acting antiretrovirals. J Acquir Immune Defic Syndr.

[53] Simoni JM, Beima-Sofie K, Wanje G, Mohamed ZH, Tapia K, McClelland RS (2021). “Lighten this burden of ours”: acceptability and preferences regarding injectable antiretroviral treatment among adults and youth living with HIV in coastal Kenya. J Int Assoc Provid AIDS Care.

[54] Gittings L, Colvin CJ, Hodes R (2022). Blood and blood: anti-retroviral therapy, masculinity, and redemption among adolescent boys in the Eastern Cape province of South Africa. Med Anthropol Q.

[55] Giles ML, Achhra AC, Abraham AG, Haas AD, Gill MJ, Lee MP (2018). Sex-based differences in antiretroviral therapy initiation, switching and treatment interruptions: global overview from the International Epidemiologic Databases to Evaluate AIDS (IeDEA). J Int AIDS Soc.

[56] Eastern Cape Department of Health. ANNUAL REPORT 2014/2015 submission of the annual report to the executive authority. 2015 [cited 2022 Jul 6]. https://provincialgovernment.co.za/department_annual/257/2015-eastern-cape-health-annual-report.pdf

[57] Brislin RW (2016). Back-translation for cross-cultural research. J Cross Cult Psychol.

[58] Pillay U, Roberts B, Rule S. South African social attitudes: changing times, diverse voices—The Human Sciences Research Council (HSRC). HSRC Press; 2006. https://www.hsrcpress.ac.za/books/south-african-social-attitudes

[59] Sherr L, Cluver LD, Toska E, He E (2018). Differing psychological vulnerabilities among behaviourally and perinatally HIV infected adolescents in South Africa-implications for targeted health service provision. AIDS Care.

[60] Slogrove AL (2018). Inequality in outcomes for adolescents living with perinatally acquired HIV in sub-Saharan Africa: a Collaborative Initiative for Paediatric HIV Education and Research (CIPHER) Cohort Collaboration analysis. J Int AIDS Soc.

[61] He E, Toska E, Cluver L, Sherr L. Determining mode of transmission amongst South African adolescents: A validation procedure using a communitytraced sample of HIV-infected adolescents in South Africa. In: 22nd International Workshop on HIV Observational Databases. 2018.

[62] Pantelic M, Boyes M, Cluver L, Thabeng M (2018). ‘They say HIV is a punishment from god or from ancestors’: cross-cultural adaptation and psychometric assessment of an HIV stigma scale for South African Adolescents Living with HIV (ALHIV-SS). Child Indic Res.

[63] Paterson DL, Swindells S, Mohr J, Brester M, Vergis EN, Squier C (2000). Adherence to protease inhibitor therapy and outcomes in patients with HIV infection. Ann Intern Med.

[64] Duong M, Piroth L, Grappin M, Forte F, Peytavin G, Buisson M (2001). Evaluation of the patient medication adherence questionnaire as a tool for self-reported adherence assessment in HIV-infected patients on antiretroviral regimens. HIV Clin Trials.

[65] Hosmer DW, Lemeshow S (2000). Applied logistic regression.

[66] Kirby T (2022). Long-acting injectable for HIV approved for use in the UK. Lancet Infect Dis.

[67] Pantelic M, Casale M, Cluver L, Toska E, Moshabela M (2022). Multiple forms of discrimination and internalized stigma compromise retention in HIV care among adolescents: findings from a South African cohort. J Int AIDS Soc.

[68] Bukenya D, Mayanja BN, Nakamanya S, Muhumuza R, Seeley J (2019). What causes non-adherence among some individuals on long term antiretroviral therapy? Experiences of individuals with poor viral suppression in Uganda. AIDS Res Ther.

[69] Bijker R, Jiamsakul A, Kityo C, Kiertiburanakul S, Siwale M, Phanuphak P (2017). Adherence to antiretroviral therapy for HIV in sub-Saharan Africa and Asia: a comparative analysis of two regional cohorts. J Int AIDS Soc.

[70] Desta AA, Woldearegay TW, Futwi N, Gebrehiwot GT, Gebru GG, Berhe AA (2020). HIV virological non-suppression and factors associated with non-suppression among adolescents and adults on antiretroviral therapy in northern Ethiopia: A retrospective study. BMC Infect Dis.

[71] UNAIDS. Addressing a blind spot in the response to HIV—Reaching out to men and boys. 2017 https://www.unaids.org/en/resources/documents/2017/blind_spot

[72] World Health Organization (2016). The use of antiretroviral drugs for treating and preventing hiv infection.

[73] Cassidy T, Grimsrud A, Keene C, Lebelo K, Hayes H, Orrell C (2020). Twenty-four-month outcomes from a cluster-randomized controlled trial of extending antiretroviral therapy refills in ART adherence clubs. J Int AIDS Soc.

[74] Mendelsohn AS, Ritchwood T (2020). COVID-19 and antiretroviral therapies: South Africa’s charge towards 90–90–90 in the midst of a second pandemic. AIDS Behav.

[75] Bailey LE, Siberry GK, Agaba P, Douglas M, Clinkscales JR, Godfrey C (2021). The impact of COVID-19 on multi-month dispensing (MMD) policies for antiretroviral therapy (ART) and MMD uptake in 21 PEPFAR-supported countries: a multi-country analysis. J Int AIDS Soc.

[76] van Staden Q, Laurenzi CA, Toska E (2022). Two years after lockdown: reviewing the effects of COVID-19 on health services and support for adolescents living with HIV in South Africa. J Int AIDS Soc.

[77] Pantelic M, Casale M, Cluver L, Toska E, Moshabela M (2020). Multiple forms of discrimination and internalized stigma compromise retention in HIV care among adolescents: findings from a South African cohort. J Int AIDS Soc.

[78] Philbin MM, Parish C, Kinnard EN, Reed SE, Kerrigan D, Alcaide ML (2020). Interest in long-acting injectable pre-exposure prophylaxis (LAI PrEP) among women in the women’s interagency HIV Study (WIHS): a qualitative study across six cities in the United States. AIDS Behav.

[79] Niehaus I, Jonsson G (2005). Dr. Wouter Basson, Americans, and wild beasts: men’s conspiracy theories of HIV/AIDS in the South African lowveld. Med Anthropol.

[80] Steinberg J. Aids and Aids treatment in a rural South African setting. 2008.

[81] Nattrass N (2013). Understanding the origins and prevalence of AIDS conspiracy beliefs in the United States and South Africa. Sociol Health Illn.

[82] Schmidt T, Cloete A, Davids A, Makola L, Zondi N, Jantjies M (2020). Myths, misconceptions, othering and stigmatizing responses to Covid-19 in South Africa: A rapid qualitative assessment. PLoS ONE.

[83] Gittings L, Casale M, Kannemeyer N, Rayalo N, Cluver L, Kelly J (2021). “Even if I’m well informed, I will never get it”: COVID-19 vaccine beliefs, intentions and acceptability among adolescents and young people in South Africa. S Afr Health Rev..

[84] El-Sadr WM, Holmes CB, Mugyenyi P, Thirumurthy H, Ellerbrock T, Ferris R (2012). Scale-up of HIV treatment through PEPFAR: a historic public health achievement. J Acquir Immune Defic Syndr.

[85] Havlir D, Gandhi M (2015). Implementation challenges for long-acting antivirals as treatment. Current Opin HIV AIDS..

[86] Minnis AM, Atujuna M, Browne EN, Ndwayana S, Hartmann M, Sindelo S (2020). Preferences for long-acting Pre-Exposure Prophylaxis (PrEP) for HIV prevention among South African youth: results of a discrete choice experiment. J Int AIDS Soc.

[87] Minnis AM, Browne EN, Boeri M, Agot K, Van Der Straten A, Ahmed K (2019). Young women’s stated preferences for biomedical HIV prevention: results of a discrete choice experiment in Kenya and South Africa. J Acquir Immune Defic Syndr.

[88] U.S. Food & Drug Administration. FDA Approves First Injectable Treatment for HIV Pre-Exposure Prevention | FDA. 2021 https://www.fda.gov/news-events/press-announcements/fda-approves-first-injectable-treatment-hiv-pre-exposure-prevention

