# Supplementary material for: Factors Associated with Preferences for Long-Acting Injectable Antiretroviral Therapy Among Adolescents and Young People Living with HIV in South Africa
Source: AIDS Behav. 2023 Jan 9;27(7):2163–75. doi: 10.1007/s10461-022-03949-2 (PMC9827015; doi:10.1007/s10461-022-03949-2)
Supplement: Supplementary file 1 — Supplementary file1 (DOCX 15 KB) [file 10461_2022_3949_MOESM1_ESM.docx]

**Additional file 1:**

**Table S1: Comparison of baseline characteristics for complete cases and lost to study follow-up (full sample)**

| Baseline Sociodemographic characteristics (N, %) | Total  (N=1 046) | Complete Cases  (N=933) | Loss-to-follow up  (N=113) | Chi-square (χ^2^) Statistic | p-value |
| --- | --- | --- | --- | --- | --- |
| Age (>15 years) | **394 (37.7)** | **337 (36.1)** | **57 (50.4)** | **8.8** | **0.003** |
| Female | 576 (55.1) | 514 (55.1) | 62 (54.9) | 0.002 | 0.964 |
| Rural residence | 271 (26.0) | 248 (26.6) | 23 (20.4) | 2.1 | 0.150 |
| Informal housing | 196 (18.8) | 172 (18.5) | 24 (21.2) | 0.5 | 0.474 |
| Double orphan | 162 (15.5) | 139 (14.9) | 23 (20.4) | 2.3 | 0.130 |
| Food insecurity | 549 (52.5) | 493 (52.8) | 56 (49.6) | 0.4 | 0.509 |
| Poverty | 692 (66.2) | 618 (66.3) | 74 (65.5) | 0.03 | 0.873 |
| Vertically acquired HIV | 824 (70.5) | 736 (70.7) | 88 (77.1) | 0.2 | 0.689 |
